# Regenerative Potential of Enamel Matrix Protein Derivative and Acellular Dermal Matrix for Gingival Recession: A Systematic Review and Meta-Analysis

**DOI:** 10.3390/proteomes9010011

**Published:** 2021-02-25

**Authors:** Muhammad Saad Shaikh, Mohid Abrar Lone, Hesham Matabdin, Muneeb Ahmed Lone, Azeem Hussain Soomro, Muhammad Sohail Zafar

**Affiliations:** 1 Department of Oral Biology, Sindh Institute of Oral Health Sciences, Jinnah Sindh Medical University, Karachi 75510, Pakistan; drsaadtanvir@gmail.com; 2 Department of Oral Pathology, Sindh Institute of Oral Health Sciences, Jinnah Sindh Medical University, Karachi 75510, Pakistan; mohid.lone@jsmu.edu.pk; 3 Department of Periodontics, Eastman Dental Institute, University College London, London WC1E 6BT, UK; hesham_87@hotmail.com; 4 Department of Prosthodontics, Dr. Ishrat-ul-Ebad Khan Institute of Oral Health Sciences, Dow University of Health Sciences, Karachi 74200, Pakistan; muneeblone@gmail.com; 5 Department of Oral Pathology, Dow University of Health Sciences, Karachi 74200, Pakistan; azeem.hussain@duhs.edu.pk; 6 Department of Restorative Dentistry, College of Dentistry, Taibah University, Al Madinah, Al Munawwarah 41311, Saudi Arabia; 7 Department of Dental Materials, Islamic International Dental College, Riphah International University, Islamabad 44000, Pakistan

**Keywords:** enamel proteome, acellular dermal matrix, alveolar bone, systematic review, periodontal regeneration

## Abstract

Objective: The purpose of this study was to assess the clinical effectiveness of using a combination of enamel matrix protein derivative and acellular dermal matrix in comparison to acellular dermal matrix alone for treating gingival recessions. Methods: The Cochrane Library (Wiley), PubMed by Medline (NLM), Medline (EBSCO), and Embase (Ovid) databases were searched for entries up to April 2020. Only clinical trials were included. Primary outcomes were root coverage (%), changes in keratinized tissue width and recession (mm). Meta-analysis was conducted for root coverage, changes in keratinized tissue width, recession, clinical attachment level and probing depth. Results: Four studies were selected for the analysis. In primary outcomes, root coverage, change in keratinized tissue width and recession analysis showed a mean difference of 4.99% (*p* = 0.11), 0.20 mm (*p* = 0.14) and 0.13 mm (*p* = 0.23) respectively between the two groups. Secondary outcomes analysis also exhibited a statistically insignificant difference between the test and control group with mean difference of 0.11 mm (*p* = 0.32) in clinical attachment level gain and -0.03 mm (*p* = 0.29) in probing depth reduction analysis. Conclusions: Within the limits of this study, enamel matrix protein derivative combined with acellular dermal matrix used for treating gingival recession defects resulted in no beneficial effect clinically than acellular dermal matrix only.

## 1. Introduction

Gingival recession and pathological loss of keratinized tissues are the most prevalent mucogingival deformities demanding surgical treatment to restore the lost supportive tissues [1]. Consequently, root coverage techniques and soft tissue augmentation are critical techniques of muco-gingival surgery protocols, including coronally advanced flaps (CAF), laterally advanced flaps (LAF), double papillae repositioned flaps, free gingival grafts (FGG), sub-epithelial connective tissue grafts (CTG), the tunnel technique as well as the pedicle flaps bilaminar technique and guided tissue regeneration or grafts [2,3,4].

Autogenous tissue grafts, particularly CTG, unquestionably remain the gold standard for root coverage treatments and soft tissue augmentation [5,6]. Considerable evidence has established that a CAF with CTG attains improved root coverage of recession [7]. However, some of the obvious drawbacks of harvesting autogenous tissue, includes post-operative bleeding and discomfort or pain at the donor site, a limited tissue supply, enhanced morbidity, much lengthier duration of surgery, and further proficiency of the surgeon [8].

To overcome such issues, various allograft substitutes (non-vital) have been investigated for plastic periodontal surgery. One of the examples is acellular dermal matrix (ADM), a human skin derivative [9]. The dermal allograft preparation includes removal of cell component and preservation of the ultrastructural integrity to prevent the inflammatory reaction [9,10,11]. Originally, ADM has been used in plastic surgery for the management of full-thickness burn wounds [10] and as an alternative to autogenous gingival grafts in root coverage procedures for last two decades [12,13,14,15,16,17]. The ADM has shown to enhance keratinized tissue, especially while treating challenging cases including thinner palatal tissues, multiple teeth recession, a restricted period of treatment, and individuals with a decreased pain threshold [12,18,19]. Moreover, ADM increased gingival thickness [14,18] and thickness of the keratinized tissue than the CAF only [16]. A histologic study suggested that the attachment of gingival tissue to the root surface was likewise for ADM and CTG, revealing epithelialization and connective tissue adherence, with the alveolar bone essentially not affected [20]. A histometric evaluation at 6-months post-operatively showed an enhanced thickness of marginal tissue, corresponding to a palatal tissue graft [20,21].

A biologic mediator, known as enamel matrix protein derivative (EMD) promotes and accelerates the regeneration of periodontal tissue (cementum, alveolar bone, and attachment tissues). Several studies including randomized controlled clinical trials (RCTs), case reports and systematic reviews (SRs) have established that EMD stimulates the new connective tissue, cementum, periodontal ligament (PDL) and bone formation for treating the periodontal osseous defects including intra-bony and furcation defects [22,23,24,25,26,27,28], periodontal reconstructive surgery [27] and root coverage [29]. For the root coverage techniques, EMD has been related to an effective root coverage, foreseeable and easy to perform with reduced patient morbidity [30,31]. In addition, EMD demonstrated positive results with regards to keratinized tissue width (KTW) and complete root coverage [32]. In contrast, few studies revealed no significant difference for the gingival recessions in the clinical results between EMD treated and non-EMD treated sites [33,34,35].

Till date, there are no definite evidence showing the extent of using the combination of ADM and different other biomolecules in terms of additional improvements of the clinical parameters compared to using the ADM alone. Furthermore, although a very few systematic reviews have been reported concerning ADM [36,37,38]; however, there are no published systematic reviews (including meta-analysis) analyzing the studies reporting combination of AMD and EMD. Hence, the extent of clinical improvements which can be achievable after the combination technique then the use of ADM alone is still debatable. Therefore, the present systematic review and meta-analysis was aimed to explore whether the addition of EMD to ADM improves the clinical outcomes with respect to root coverage (RC), KTW and gingival recession (REC) reduction compared to using the ADM alone.

## 2. Methodology

The present systematic review was conducted using the PRISMA (Preferred Reporting Items for Systematic Reviews and Meta-analysis) principles [39] and compared the clinical efficacy of EMD + ADM and ADM in treating gingival recession defects. 

### 2.1. Focused Question

The focused question was devised based on the population intervention control outcome (PICO) principle. The focused question for this study was, “*What are the clinical benefits of using a combination of enamel matrix derivative (EMD) and acellular dermal matrix (ADM) with coronally advanced flap (CAF) for the treatment of gingival recession lesions*”.

The PICO was designed as:

Population (P): Gingival recession defects (Millers class I to III)

Intervention types (I): CAF + EMD + ADM

Comparison/control (C): CAF + ADM

Outcome measures (O): Primary outcomes → Percentage of RC, change in KTW and REC reduction. Secondary Outcomes → Clinical attachment level (CAL) gain, pocket depth (PD) reduction

### 2.2. Literature Search

The literature search was performed on electronic databases until April 2020 using Cochrane Library (Wiley), PubMed (NLM), Medline (Ovid) and Embase (Ovid) databases. Following keywords (italics) with Boolean operators were used for search process, P: “*gingival recession*” OR “*recession defect*” OR “*root coverage*” AND I: “*enamel matrix derivative*” OR “*enamel matrix proteins*” OR “*EMD*” OR “*acellular dermal matrix allograft*” OR “*acellular dermal matrix*” OR “*ADM*” OR “*allograft*” AND C: “*coronally advanced flap*” OR “*CAF*” OR “*coronally positioned flap*” OR “*CPF*” OR “*surgical flaps*” AND O: “*keratinized tissue width*” OR “*complete root coverage*”. In addition, the Open Grey (www.opengrey.eu, accessed on 3 March 2021) was used for searching the Grey literature. The reference list of related articles was hand searched for more studies. Furthermore, the Periodontology specialty journals were also searched. In the case of ambiguous or missing data, experts were contacted directly.

### 2.3. Literature Selection

Criteria for including a study was as follows:

- Controlled Clinical Trials (CCTs) or RCTs in which combination of EMD and ADM is compared to ADM only.
- Millers class I to III gingival recession of ≥2 mm (measured from cemento-enamel junction to the gingival margin).
- Mean follow-up period of ≥6-months.

The exclusion criteria included:

- Class IV gingival recession.
- Laboratory or animal-based (in vitro and histologic) studies
- Reviews or systematic reviews, case series and case reports studies.
- Studies other than in English Language.

### 2.4. Literature Screening 

A systematic screening of the retrieved articles was carried out by two independent researchers (MSS and MAL) in three stages. In the first stage, researchers independently read the retrieved titles and keywords to assess the fulfillment of the inclusion criteria. In the second stage, the abstracts were carefully screened for their relevance to the research question. In the third stage, the full-text of the screened articles were retrieved and meticulously analyzed according to the eligibility criteria. All the included studies were analyzed for the primary (RC (%), change in KTW (mm) and REC reduction (mm)) and the secondary (CAL gain (mm), PD reduction (mm)) outcome measures. The kappa coefficient (***κ***) was used to analyze inter-rater agreement between the researchers [40]. In case of any discrepancy, consent was be obtained by discussion with a third reviewer (MSZ).

### 2.5. Assessment of Quality and Bias Risk

In regard to RCTs and CCTs, the Cochrane risk of bias tool [41] was applied to perform the quality assessment, analyzing different domains including:

- Selection bias
- Performance bias
- Detection bias
- Attrition bias
- Reporting bias and
- Other types of bias

### 2.6. Data Analysis

The homogeneity of outcomes between the studies allowed the quantitative synthesis by meta-analysis using the Review Manager 5.3. (Cochrane Collaboration, Oxford, UK) for MacOS software. For primary and secondary outcomes, mean differences along with 95% confidence intervals of differences (95% CI) were estimated. A *p* value of less than 0.05 was regarded to be of statistical significance. The forest plot to elucidate the weighted mean difference (WMD) of the outcome in every study as well as the final estimate was used. Based on the assumption of a population of studies with probable variations, a random-effects model (inverse-variance) was implemented. Heterogeneity resultant from the discrepant treatment effect of each paper was calculated via the Cochran’s test [42]. The outcomes of the I^2^ test (low: 25%; moderate: 50%; high heterogeneity: 75%) and Q statistic (significant at *p* < 0.10) were applied to measure the heterogeneity level [43]. Sensitivity analyses as well as subgroup analyses were conducted where indicated.

### 2.7. Publication Bias

The meta-analyses with fewer than 10 research papers were not assessed, contemplating the power to detect the publication bias [44]; else funnel plots were used to assess the publication bias [45].

## 3. Results

**Figure 1:**
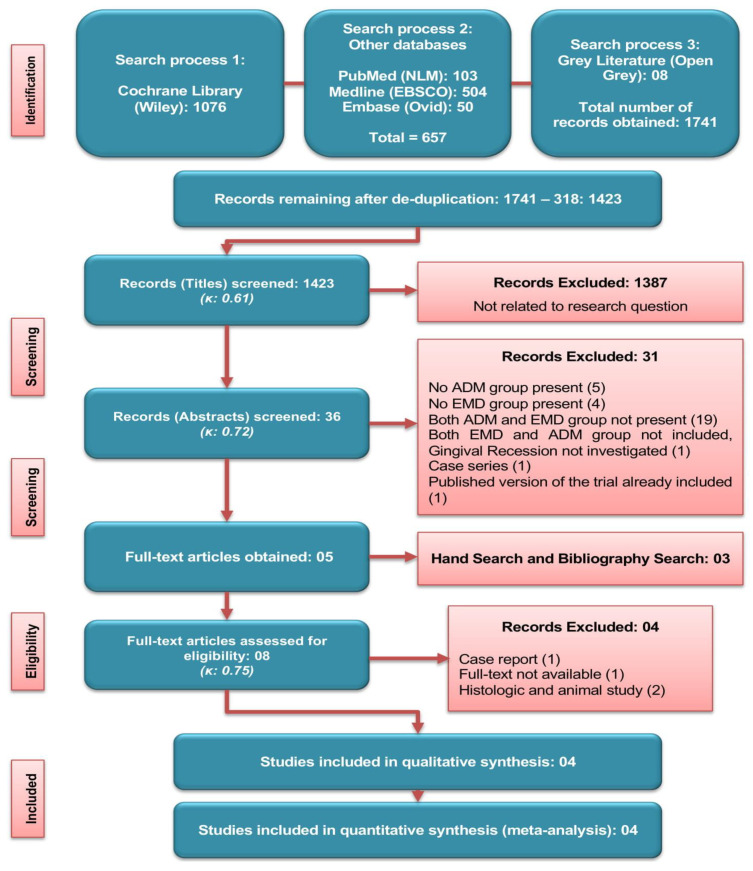
Literature search strategy and screening criteria used.

The screening and search results are shown in Figure 1. Out of 1423 titles, five studies were selected for the full-text evaluation. A further manual search added three more articles, making a total of eight papers assessed for the eligibility. Four studies were excluded for not fulfilling the selection criteria [animal and histologic studies (*n* = 2), case report (*n* = 1), full-text not available (*n* = 1)] (Table 1), and four RCTs were finally included for the quantitative as well as qualitative analysis (Figure 1). 

From the 31 excluded abstracts, 22 papers used several different sources of soft tissue grafting (supplemental figure) such as autogenous [10 papers (45%)], xenografts [2 papers (9%)], synthetic graft [one paper (5%)], and combined sources [8 papers (36%)]. Only one paper (5%), used two allografts including ADM, but due to no EMD interventional group, this study was excluded.

### 3.1. Study Characteristics

The study characteristics of papers included are demonstrated in Table 2. All the four RCTs [46,47,48,49] were single-center, split-mouth randomized controlled clinical trials, used antibiotics and were conducted in the institutional setup. Furthermore, in each study, the intervention group used EMD combined with ADM, where the control group used only ADM [46,47,48,49]. The follow-up time period varied between the included RCTs (i.e., 3- and 6-months [46], 2-, 4- and 6-months [47], 6-months [48] and 3-, 6- and 12-months [49]). The comparatively shorter follow-up time periods, especially for Shin et al. [46] and Pourabbas et al. [47], are one of the limitations of these studies. 

The extent of inter-rater reliability between the two reviewers was tested using the Cohen’s Kappa coefficient. The calculated scores of Cohen’s Kappa statistic ***κ*** were 0.61 0.72 and 0.75 indicating substantial to almost perfect agreement [40].

**Table 1:** Excluded studies after full-text analysis.

Author/s	Reason for Exclusion
Saadoun [50]	Case report
Wallace [51]	No full-text available
de Oliveira et al. [52] Shirakata et al. [53]	Histologic and animal study

**Table 2:** Characteristics of the studies.

Authors	Study Design	Treatment		Antibiotics	Follow-Up (Months)
		Test Group	Control Group		
Shin et al. [46]	Single-center Split-mouth RCT Prospective	EMD + ADM + CAF	ADM + CAF	Y	3, 6
Pourabbas et al. [47]	Single-center Split-mouth RCT Prospective	EMD + ADM + CAF	ADM + CAF	Y	2, 4, 6
Alves et al. [48]	Single-center Split-mouth RCT Prospective	EMD + ADM + CAF	ADM + CAF	Y	6
Costa et al. [49]	Single-center Split-mouth RCT Prospective	EMD + ADM + CAF	ADM + CAF	Y	3, 6, 12

ADM (Acellular dermal matrix), CAF (Coronally advanced flap), EMD (Enamel matrix protein derivative), RCT (Randomized controlled clinical trial), Y (Yes).

### 3.2. Participant and Defect Characteristics

A total of 58 patients with a range of age between 23 and 63 years were reported from the included studies. Only one study reported the mean age of 45.4 years [46]; however, three studies [47,48,49] did not report the mean age. Three studies reported the number of males and females [46,47,48], whereas one study did not mention the gender of the participants [49]. Three studies included the smokers [46,48,49] and one study did not include the smokers [47]. One dropout was reported in one study [48] with reason, while there were no drop-outs in other three included studies [46,47,49] (Table 3). The studies reported a total of 194 defects (ranging from 36 to 82). In three papers, class I and II gingival recession lesions (Millers) were cured [47,48,49], whereas in one publication, Miller class I to III gingival recession defects were selected [46]. Furthermore, in every study, anterior and premolars were the type of tooth treated (Table 3).

### 3.3. Quality Assessment of Included Trials

**Figure 2:**
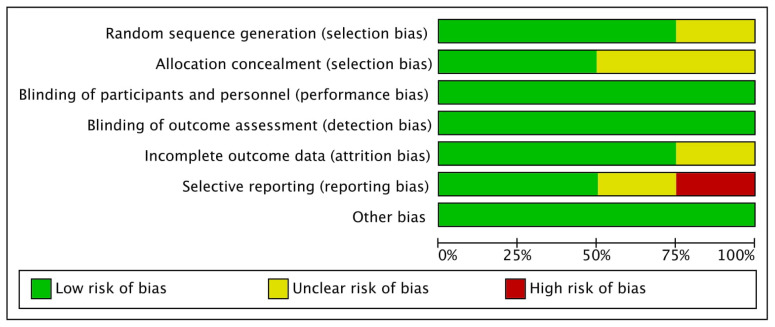
Bias risk graph: Individual risk of bias item shown as percentages across each included paper.

**Figure 3:**
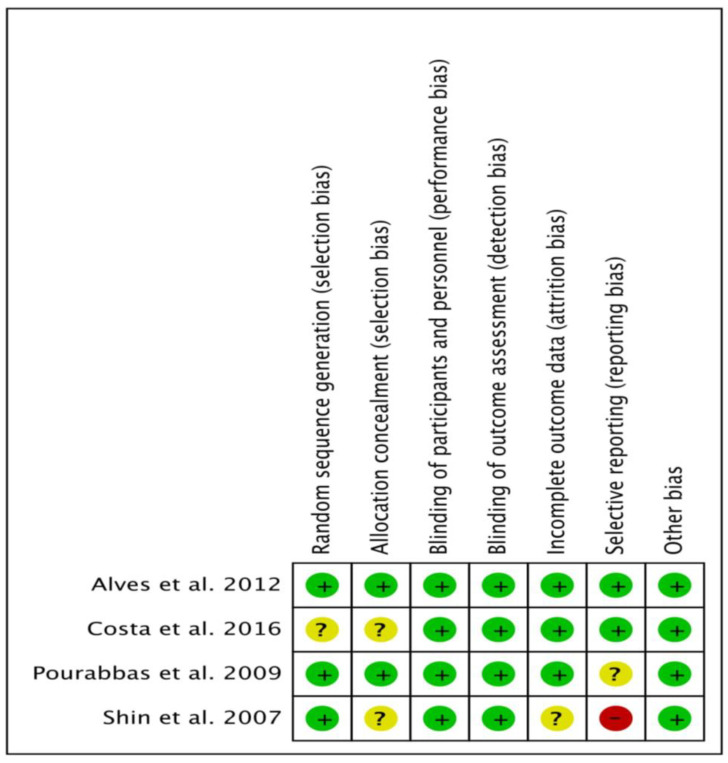
Bias risk summary: Each risk of bias item for the included papers.

Figure 2 shows percentages across every included paper for individual risk of bias item, whereas Figure 3 demonstrates risk of bias item for every included study. Out of four studies, one RCT each was categorized as low risk [48] and high risk [46] of bias, and two RCTs [47,49] were categorized as unclear risk of bias.

### 3.4. Effects of Primary and Secondary Outcomes

**Figure 4:**
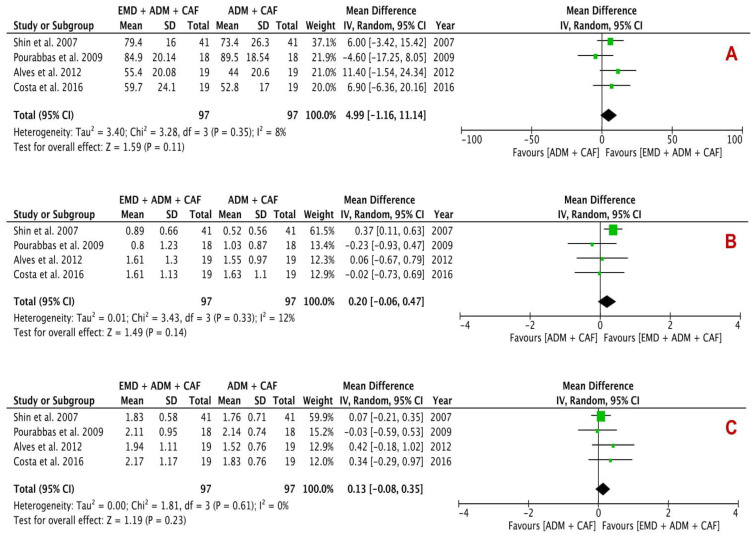
Forest plots evaluating primary outcomes (**A**) RC, (**B**) Change in KTW and (**C**) REC reduction following surgical treatment using EMD + ADM and ADM alone (random effects; 95% CI; weighted mean difference).

**Figure 5:**
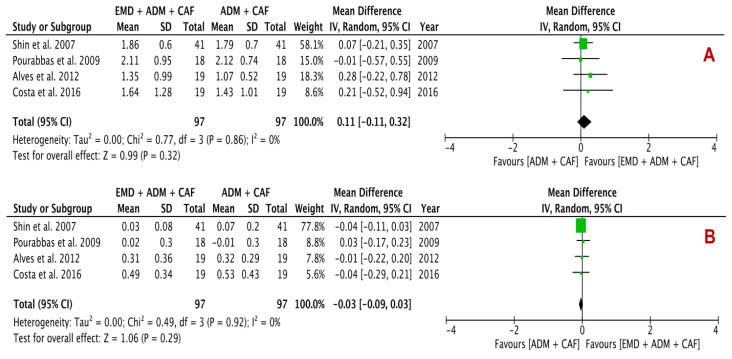
Forest plots evaluating secondary outcomes (**A**) CAL gain and (**B**) PD reduction following surgical treatment using EMD + ADM and ADM alone (random effects; 95% CI; weighted mean difference).

Clinical changes in terms of RC (%), KTW (mm), REC (mm) (primary outcomes), and CAL (mm) and PD (mm) (secondary outcomes) are summarized in Table 4. Quantitative analysis of three primary outcomes is shown in Figure 4 and two secondary outcomes is revealed in Figure 5. Four studies each were included in every analysis. 

In primary outcomes, RC, change in KTW and REC analysis showed a mean difference of 4.99% (*p* = 0.11; 95% CI −1.16–11.14), 0.20 mm (*p* = 0.14; 95% CI −0.06–0.47) and 0.13 mm (*p* = 0.23; 95% CI −0.08–0.35) respectively between the experimental group and control group, indicating no beneficial effect of EMD + ADM than ADM. The analysis of RC and change in KTW showed low percentage of heterogeneity with I^2^ value of 8% and 12% respectively; however, change in KTW analysis demonstrated no heterogeneity (I^2^: 0%).

The secondary outcomes analysis included CAL gain and PD reduction, presenting a statistically insignificant change between the EMD + ADM and ADM alone with difference in mean of 0.11 mm (*p* = 0.32; 95% CI −0.11–0.32) in CAL gain and −0.03 mm (*p* = 0.29; 95% CI −0.09–0.03) in PD reduction analysis. The I^2^ value was found to be 0% for both analyses, suggesting no heterogeneity.

## 4. Discussion

This systematic review estimated the clinical efficacy of using either ADM alone or EMD and ADM combination for the regenerative periodontal surgery of gingival recession defects. For this purpose, various primary [RC (%), change in KTW (mm) and REC reduction (mm)] and secondary [CAL (mm) and PD (mm)] parameters for gingival recession defects were evaluated. The quantitative analysis using forest plots (Figure 4 and Figure 5) demonstrated a statistically insignificant difference between the test group (EMD + ADM) and control group (ADM only) (*p* < 0.05). Only two forest plots (Figure 4) showed low heterogeneity, whereas other forest plots revealed no heterogeneity, suggesting little variability between the studies. All the included studies conducted qualitative and quantitative analysis using the split-mouth designs. A fundamental benefit of this design of study is that each individual act as his/her own control and hence the variability between the subjects is almost eliminated therefore enhancing the study power. In addition, the requirements of sample size for this study design is around half than that of a parallel study design [54].

In terms of smoking history, three studies included smokers [46,48,49], whereas one study include only non-smokers [47]. Further studies are needed to differentiate smokers and non-smokers to adequately evaluate the efficacy of these treatment modalities. All the four included studies used postoperative antibiotics; therefore, the beneficial effects of postoperative antibiotics have not been evaluated. It is plausible that the use of postoperative antibiotics may act as a confounding factor [55,56].

Histological studies on recession defects using EMD combined with CAF [57], CTG [58,59] barrier membranes [60,61] revealed tissue formation essential for periodontal regeneration including cementum, PDL fibers and alveolar bone. An RCT using CAF plus EMD showed that it is a predictable therapeutic modality for gingival recession defects, easy to implement, low morbidity and a significant increase in the RC percentage, change in KTW and CAL [62]. In a paper assessing the role of EMD in the surgical management of recession defects, Cheng et al. (2015) in a SR showed that CAF + CTG and CAF + EMD significantly improved KTW compared to the CAF alone. Using EMD reduced PD; however, the difference was insignificant [63]. Modica et al. (2000), Discepoli et al. (2019) and Górski et al. (2020) showed that there is no difference between EMD treated gingival recessions and non-EMD treated gingival recessions with respect to the clinical outcomes [64,65,66]. In another study, this biologically active regenerative material increased the percentage of RC and KTW than the control group [67]. The present review validated that both ADM + EMD and ADM only enhanced the clinical variables considerably; however, between the two treatments, there was no statistically significant difference. Besides, a histological study [52] found that EMD does not contribute to improved cementum and bone regeneration when associated with ADM. On the contrary, another histological study [53] demonstrated better cementum and bone regeneration for EMD + ADM group as compared to ADM only and CAF only groups.

The study hypothesis was that the EMD might increase the potential of wound healing due to its potential of stimulating angiogenesis [68,69] and fibroblast activity [70,71] when applied to the interface of ADM-soft tissue. Additionally, EMD may also play a role in the propagation of PDL cells, increase formation of proteins including collagen, and promote mineralization [30,70,71] when used at the ADM-root surface interface. Another study exhibited that EMD is found at the site of healing during majority of the essential phases occurring during wound healing of the gingival tissues [72,73].

Cummings et al. (2005), reported the management of teeth with severe gingival recessions (Miller Class III/IV) using a combination of CAF and ADM. Formation of cementum in the apical part of the recession defects was observed with no signs of novel osteogenesis. Additionally, ADM adherence to the surface of root was accomplished via the formation of both long junctional epithelium as well as connective tissue adherence however there were no signs of PDL fibers entrance to the surface of root [20]. Inductive effects of EMD on the periodontium components; predominantly fibroblasts have been reported by in vitro studies [74,75], while the in vivo studies suggest controversial findings [64,76]. This can be attributed to technique sensitiveness of EMD application leading to change in outcomes and disagreement. The application of ADM may lead to EMD dispersion and emission from the wound area as a result of its physical pressure and effects on thickness of flap as well as tension. In contrast, ADM permeability to EMD has not been confirmed and ADM presence may disrupt the interaction between fibroblasts and EMD. In addition, ADM on its own may stimulate proliferation of cells and ultrastructural cellular changes, accredited to the presence of extracellular materials [14] and leading to an improved inductive role of ADM on EMD.

A routine encounter for root coverage surgical techniques is the graft’s ability to persist at the surgical receptor site. Especially when dealing with a non-vital ADM graft, this must be contemplated as an even more critical factor [77]. Studies investigated variations in gingival as well as alveolar mucosa microcirculation after various surgical flaps or incisions in a fluorescein angiographic evaluation [78] and laser Doppler flowmetry [79,80] the flaps must be wide enough at their base to comprise the main vessels of gingival tissue. The vertical releasing incisions should never be in close proximity to tooth with gingival recession defect. Since the principle gingival vascular supply is directed caudocranially to the gingival margin from the vestibule [81], a wider flap enhances the amount of blood vessels accessible to contribute in the process of healing. The modified technique [82] was used in two studies [48,49] and based on the extension of surgical site to adjacent teeth with the intention to give more nutrients, vascular supply, and an enhanced source of cells to support the ADM absorption. A wider flap also permitted for much improved tissue manipulation, particularly for attaining a tension free CAF to completely cover the allograft [82,83]. Whereas, Shin et al. (2007) used the incision design by Zucchelli and Sanctis [84] for recession defects involving multiple teeth, eluding vertical releasing incisions. Therefore, healing of several root coverage procedures and the impact of some other variables including thickness of flap, the coronal flaps tension and vestibular depth on the outcomes imply a compulsion for more human, animal, or histological studies to explore their plausible effect as prognostic factors. Nevertheless, ADM is the most widely studied soft tissue allograft till date than any other allografts and it has already provided very favorable results in general plastic surgeries. Comparing ADM to autogenous grafts, it prevents the need for second surgical site and consequently post-operative pain and discomfort [8].

In the present review, two studies revealed unclear risk of allocation concealment, which can introduce selection bias. Furthermore, one study showed high risk of selective reporting, indicating presence of reporting bias. Overall, quality appraisal of the selected papers in the meta-analysis were mostly categorized as unclear risk. The choice to include each study in the meta-analysis was due to scarcity of data in the literature as only four RCTs could be identified according to the exhaustive literature search. Moreover, the results of this study should be interpreted cautiously due to the fact that only four RCTs were included after the exhaustive search, not providing a very strong evidence. Other limitations of this review were shorter follow-up time periods and small sample sizes of the studies included in the quantitative analysis. Therefore, for future research, more interventional studies particularly RCTs with much bigger sample sizes and longer-term follow-ups are required for further validation of the effect of combination therapy of EMD + ADM against ADM alone in gingival recession defects.

Furthermore, the availability of data from the clinical trials (mostly Class I/II gingival recession cases) could be a limitation to conclude for this application setting and therefore, future research should also emphasize on these limitations and directing the lack of evidence for severe recession cases. 

## 5. Conclusions

Enamel matrix protein derivative and acellular dermal matrix are commonly explored for gingival recessions regenerative therapy. Using a combination of EMD and ADM for treating gingival recession defects resulted no remarkable beneficial effects compared to ADM only in terms of RC, change in KTW, REC, CAL, and PD statistically. Accordingly, both treatments can serve as an alternate to root coverage of Millers class I and II gingival recession lesions. However, the cost-benefit ratio associated with adding EMD to ADM procedure should be carefully evaluated.

## Figures and Tables

**Table 3:** Characteristics of participants and defects.

Authors	Participant Characteristics						Defect Characteristics		
	*n*	Mean Age (Years)	Age Range (Years)	Gender (M/F)	Drop-Outs	Smoking	Number of Defects	Defect Type	Type of Tooth
Shin et al. [46]	14	45.4	23–62	8/6	0	In	82	Miller I to III	Non-molars
Pourabbas et al. [47]	15	NR	26–63	7/8	0	Ex	36	Miller I and II	Non-molars
Alves et al. [48]	20	NR	30–50	7/12	1	In	38	Miller I and II	Non-molars
Costa et al. [49]	19	NR	30–50	NR	0	In	38	Miller I and II	Non-molars

Ex (Excluded), In (Included), NR (Not reported).

**Table 4:** Effects of intervention and evaluation of various parameters.

Authors	RC (%)	KTW Change (mm)	REC Change (mm)	CAL Change (mm)	PD Change (mm)
Shin et al. [46]	T: 79.4 ± 16.0	T: 0.89 ± 0.66	T: 1.83 ± 0.58	T: 1.86 ± 0.60	T: 0.03 ± 0.08
	C: 73.4 ± 26.3	C: 0.52 ± 0.56	C: 1.76 ± 0.71	C: 1.79 ± 0.70	C: 0.07 ± 0.20
Pourabbas et al. [47]	T: 84.90 ± 20.14	T: 0.8 ± 1.45	T: 2.11 ± 1.37	T: 2.11 ± 0.95	T: 0.02 ± 0.30
	C: 89.5 ± 18.54	C: 1.03 ± 1.53	C: 2.14 ± 1.42	C: 2.12 ± 0.74	C: -0.01 ± 0.30
Alves et al. [48]	T: 55.4 ± 20.08	T: 1.61 ± 1.30	T: 1.94 ± 1.11	T: 1.35 ± 0.99	T: 0.31 ± 0.36
	C: 44 ± 20.6	C: 1.55 ± 0.97	C: 1.52 ± 0.76	C: 1.07 ± 0.52	C: 0.32 ± 0.29
Costa et al. [49]	T: 59.7 ± 24.1	T: 1.61 ± 1.13	T: 2.17 ± 1.17	T: 1.64 ± 1.28	T: 0.49 ± 0.34
	C: 52.8 ± 17.0	C: 1.63 ± 1.10	C: 1.83 ± 0.76	C: 1.43 ± 1.01	C: 0.53 ± 0.43

C (Control group), CAL (Clinical attachment level), RC (Root coverage), KTW (Keratinized tissue width), PD (Probing depth), REC (Recession), T (Test group).

## Data Availability

Data supporting the reported results can be found at Cochrane Library (Wiley), PubMed by Medline (NLM), Medline (EBSCO) and Embase (Ovid) databases.
